# Partial Least Square Aided Beamforming Algorithm in Magnetoencephalography Source Imaging

**DOI:** 10.3389/fnins.2018.00616

**Published:** 2018-09-05

**Authors:** Yegang Hu, Chunli Yin, Jicong Zhang, Yuping Wang

**Affiliations:** ^1^School of Biological Science and Medical Engineering, Beihang University, Beijing, China; ^2^Beijing Advanced Innovation Centre for Big Data-Based Precision Medicine, Beihang University, Beijing, China; ^3^Beijing Advanced Innovation Centre for Biomedical Engineering, Beihang University, Beijing, China; ^4^Hefei Innovation Research Institute, Beihang University, Hefei, China; ^5^Department of Neurology, Xuanwu Hospital, Capital Medical University, Beijing, China; ^6^Beijing Key Laboratory of Brain Functional Disease and Neuromodulation, Beijing, China

**Keywords:** Magnetoencephalography (MEG), beamforming, partial least squares, source imaging, epileptogenic zone, imaging-based marker

## Abstract

Beamforming techniques have played a prominent role in source imaging in neuroimaging and in locating epileptogenic zones. However, existing vector-beamformers are sensitive to noise on localization of epileptogenic zones. In this study, partial least square (PLS) was used to aid the minimum variance beamforming approach for source imaging with magnetoencephalography (MEG) arrays, and verified its effectiveness in simulated data and epilepsy data. First, PLS was employed to extract the components of the MEG arrays by maximizing the covariance between a linear combination of the predictors and the class variable. Noise was then removed by reconstructing the MEG arrays based on those components. The minimum variance beamforming method was used to estimate a source model. Simulations with a realistic head model and varying noise levels indicated that the proposed approach can provide higher spatial accuracy than other well-known beamforming methods. For real MEG recordings in 10 patients with temporal lobe epilepsy, the ratios of the number of spikes localized in the surgical excised region to the total number of spikes using the proposed method were higher than that of the dipole fitting method. These localization results using the proposed method are more consistent with the clinical evaluation. The proposed method may provide a new imaging marker for localization of epileptogenic zones.

## Introduction

Accurate diagnosis of epileptogenic zones has long been a focus of neurology, as it determines whether epilepsy patients can achieve seizure freedom by surgical excision. In recent years, magnetoencephalography (MEG) has been increasingly trusted by clinical epileptologists for preoperative examination (Wennberg and Cheyne, 2014; Nissen et al., 2016). This is because MEG is a non-invasive neuroimaging technique that records brain activity with millisecond temporal resolution and minor signal deterioration from the skull and scalp (Barnes and Hillebrand, 2003; Zumer et al., 2007; Baillet, 2017). Postsynaptic current flow within the dendrites of active neurons produces a weak magnetic field that can be measured by superconducting quantum interference devices (SQUIDS) (Hämäläinen et al., 1993). Because the currents generated by neurons determine the magnitude of the measured fields, these measurements can give information about brain activity on a millisecond time scale. However, the great challenge is to localize the active neurons on the basis of the measured magnetic field. Because the locations of epileptogenic zones estimated using current localization methods are not always accurate, MEG has not been completely accepted by all clinical epileptologists (Englot et al., 2015). In general, the number of sensors is far less than the number of possible current distributions. This inverse problem is an example of what mathematicians call an ill-posed problem (Hadamard and Morse, 1953). Finding the optimal solution of such an underdetermined system of equations often requires specific constraints. In other words, to accurately localize the brain sources of magnetic signals, assumptions must be made about the nature of the neuronal sources.

Many source imaging algorithms for MEG signals have been proposed (Pascual-Marqui et al., 1994; Mattout et al., 2006; Grech et al., 2008; Mäkelä et al., 2018), and each optimizing the solution of the inverse problem under a specific set of assumptions. One type of inverse solution approach is known as “beamforming” (Van Veen et al., 1997; Groß et al., 2001; Sekihara et al., 2002a; Oshino et al., 2007; Zhang and Liu, 2015). Beamforming techniques play a key role in signal processing and neuroimaging. These methods make use of spatial filtering, that is, the MEG signals are decomposed into “beams” based on gain vectors corresponding to specific source-grid points (Diwakar et al., 2011). The most widely used beamforming method, linearly constrained minimum variance (LCMV) beamforming (also called vector beamforming), produces a reliable spatial filter when the weights are chosen to minimize the filter output power subject to a linear constraint. However, the existing vector beamformers for MEG source imaging are sensitive to noise, and poor at localizing sources. The main reason for this problem is that the sensor array geometry is used directly to estimate the covariance matrix. Recently, an iterative spatiotemporal signal decomposition method has been used to modify the vector beamforming technique, and has been successfully applied to source localization for MEG signals (Hu et al., 2017). Although the approach has achieved high spatial accuracy, the correlations of signals from different brain regions are ignored when the components of the MEG arrays are extracted. An improvement would be to use partial least squares (PLS) analysis to make better use of structural information.

PLS analysis originated in the fields of econometrics and chemometrics (Wold et al., 1984; Geladi and Kowalski, 1986). It extracts components in a way that maximizes the covariance between each component and a “class variable.” In recent years, this approach has been successfully applied in many fields, including multivariate statistics (Wold et al., 1984), analytical chemistry (Wold et al., 2001), face recognition (Baek and Kim, 2004; Sharma and Jacobs, 2011), and bioinformatics (Boulesteix and Strimmer, 2006). In pattern recognition, the PLS method can be used to extract the principle components with maximum variability and to exploit the class information (Baek and Kim, 2004; Sharma and Jacobs, 2011). PLS has better performance in feature extraction and denoising compared with typical methods, such as principal component analysis (PCA) and linear discrimination analysis (LDA). The principal components extracted by PLS are called “intrinsic components” to indicate that PLS is more representative for biometric signals. In fact, MEG signals are very similar to these biometric signals, and are affected by various noises. If the intrinsic components are found from those MEG signals, these components should be usable to improve the spatial accuracy of source imaging. Thus, in this study we used the PLS method to extract the components of the MEG signals and reconstruct the data matrix in this study. Although recent literatures show that the PLS method has been used in functional neuroimaging (McIntosh and Lobaugh, 2004; Krishnan et al., 2011; Cheung et al., 2016), for purposes such as describing the relationship between brain activity and behavior, these PLS applications are not intended to improve the source imaging method.

The aim of this study was to propose and investigate a new source imaging algorithm with specific applicability to focal epilepsy focus localization. We applied PLS analysis to aid the vector beamforming technique for better performance in source imaging with MEG arrays. First, the MEG arrays were treated as an observation matrix ***X***, combined with a class matrix ***Y*** of dummy variables that code for brain regions. Second, we employed PLS technique to extract the components of the MEG arrays by maximizing the covariance between a linear combination of the predictors and the class variable. We then reconstructed the sensor arrays based on the components and loadings, and used the vector beamforming technique to estimate the source model. The newly proposed source imaging approach for MEG recordings was first validated on simulated data, and compared with three other well-known beamforming methods, linearly constrained minimum variance (LCMV) (Van Veen et al., 1997), dynamic imaging of coherent sources (DICS) (Groß et al., 2001), and modified LCMV with iterative matrix decomposition (mLCMV) (Hu et al., 2017). Since these methods belong to the beamforming family, the basic assumption in this study is the same as assumption underlying the minimum variance beamforming. We further verify the proposed method in a real dataset that includes the MEG recordings of 10 patients with temporal lobe epilepsy.

## Methods

### Partial least squares analysis

PLS analysis is a technique for extracting components and loadings between a set of input variables ${\left\{x_{i}\right\}}_{i=1}^{M}\in R^{N}$ and a set of response variables ${\left\{{\text{y}}_{i}\right\}}_{i=1}^{M}\in R^{L}$. As with principal components analysis, PLS generates uncorrelated components that are linear are linear combinations of the original input variables. The difference is that PLS creates the components by modeling the relationship between the input and response variables, while maintaining most of the information in the input variables. The objective criterion is to find a weight vector ***w***_*k*_ such that

(1) $$\begin{array}{r} w_{k}=\arg\underset{||w||=1,||\upsilon ||=1}{\max}{\text{Cov}}^{2}\;\left(Xw,\;Yv\right)\\ subject\;to\;the\;constraint\;w_{k}^{T}{Cw}_{j}=0 \end{array}$$

where ||•|| denotes the two-norm operator, 1 ≤ *j* ≤ *k*, and *Cov* (∙, ∙) *Cov* (•, •) represents the covariance operator, T is the transpose of a vector or matrix, and ***C* = *X***^*T*^ ***X***. Here, ***X*** represents an *M* × *N* matrix of input variables and ***Y*** is an *M* × *L* (*M* × *L*) matrix made up of corresponding response variables. In general, the column vectors of the data matrices ***X*** and ***Y*** are normalized before optimization, that is, the mean value is zero and the variance is one.

Next, a well-known iterative algorithm (Lewi, 1995; Rosipal and Trejo, 2001; Hu et al., 2012) is used to optimize formula (1). A description in pseudo-code is shown in Algorithm 1:

**Algorithm 1:**
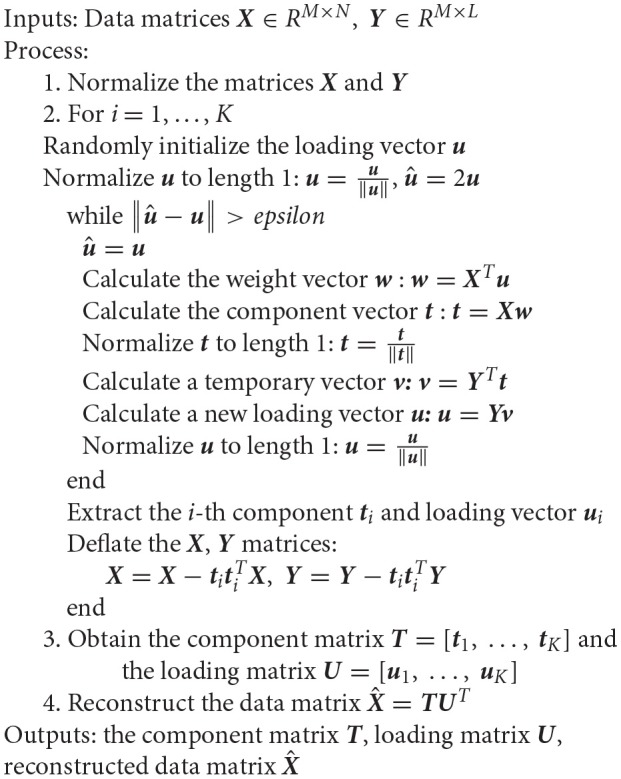
**Pseudo-code for partial least squares analysis**.

Note that the variable *K* in Algorithm 1 is determined by the two-norm of the residual matrix of the data matrix ***X***, that is, as long as the norm value is greater than a threshold, the program continues to cycle. In addition to being used for data reconstruction, PLS analysis can also be used effectively for dimensionality reduction, recognition, and regression (Rosipal and Trejo, 2001; Baek and Kim, 2004; Hu et al., 2012).

In feature extraction and pattern classification, PLS is a supervised learning method, and each row in the ***Y*** matrix is a class label for each sample. PLS is then used to extract the intrinsic components ***T*** and loadings ***U*** by using Algorithm 1, and the data matrix $\hat{X}$ is reconstructed using these intrinsic components and loadings. The new data matrix $\hat{X}$ is a relatively clean matrix after denoising. This procedure can be considered to improve the spatial accuracy of source imaging. The ***Y*** matrix is very important and will be described in Section Source Imaging via Partial Least Squares.

### Minimum variance beamforming

Beamforming, also called spatial filtering, plays an important role in localizing sources of brain activity from surface recordings. The weights of the spatial filter are usually obtained by minimizing the filter output power (i.e., minimizing the variance). LCMV beamforming optimizes the objective function subject to a linear constraint, and therefore is a type of vector beamforming. The principle of using minimum variance beamforming to solve inverse problems will now be illustrated in detail. For an input variable set ***X*** = [***x***^(1)^, ***x***^(2)^, …, ***x***^(*N*)^ ], derived from the MEG sensors, the inverse solution model is given as:

(2) $$\begin{array}{r} X=LD+\varepsilon \end{array}$$

where ***x***^(*i*)^ represents an *M* × 1 vector of the MEG recordings at the *i*-th time point (*i* = 1 ⋯ *N*), *M* is the number of MEG sensors, ***L***is the *M* × *J* (lead-field) gain matrix, *J* is the number of unknown dipole moment parameters, ***D*** denotes a *J* × *N* dipole moment matrix for a given time series, *N* is the number of time points, and **ε*** represents the *M* × *N* noise matrix. We design a spatial filter ***W*** (*r*_0_) for the narrowband volume element centered on location *r*_0_, using the following formula:*

(3) $$\begin{array}{r} y=W^{T}\;\left(r_{0}\right)\;x \end{array}$$

where ***W*** (*r*_0_) is an *M* × 3 matrix, ***x*** represents the input vector of the filter, and ***y*** is the output vector. Generally, an ideal narrowband spatial filter needs to satisfy

(4) $$W^{T}\;\left(r_{0}\right)\;L\;\left(r\right)=\{\begin{array}{c} I,\;r=r_{0}\\ 0,\;r\neq r_{0} \end{array}$$

where *r* is the location of a grid point inside the brain, ***L*** (*r*) is the *M* × 3 (lead-field) gain matrix, and ***I*** is the unit matrix. The objective function to be optimized is then posed mathematically as

(5) $$\begin{array}{r} \begin{array}{c} \underset{W\;\left(r_{0}\right)}{\min}\;tr\;\left(W^{T}\;\left(r_{0}\right)\;C\;\left(x\right)\;W\;\left(r_{0}\right)\right)\;\\ subject\;to\;W^{T}\;\left(r_{0}\right)L\left(r_{0}\right)=I\; \end{array} \end{array}$$

where *tr*(•) denotes the trace of a matrix, and ***C*** (***x***) is the covariance matrix of random variables based on the row vectors of the data matrix ***X***. A second-order statistic for the sample is used to estimate the population covariance, as illustrated in the study by (Van Veen et al., 1997). The effect of the constraint here is to allow the activity at position *r*_0_ to be passed with unit gain, while inhibiting contributions from all other sources.

An algorithm that minimizes interference (MinInf) is used to optimize the objective function, yielding the optimal solution (Groß and Ioannides, 1999)

(6) $$\begin{array}{r} W^{T}\;\left(r_{0}\right)\;=\;{\left[L^{T}\;\left(r_{0}\right)\;C^{-1}\left(\text{x}\right)\;L\left(r_{0}\right)\right]}^{-1}L^{T}\;\left(r_{0}\right)\;{\text{C}}^{-1}\left(\text{x}\right) \end{array}$$

where (•)^−1^ denotes the inverse operator. The formula (6) is substituted into the spatial filter, and the variance of the filter output is estimated to be:

(7) $$\begin{array}{r} \hat{V}ar\;\left(r_{0}\right)\;=\;tr\;\left\{{\left[L^{T}\;\left(r_{0}\right)\;C^{-1}\;\left(\text{x}\right)\;L\;\left(r_{0}\right)\right]}^{-1}\right\} \end{array}$$

The estimated variance is the value of the objective function (5) at its minimum, or it can represent the strength of the activity at grid point *r*_0_. Therefore, if the MEG data matrix ***X*** is known, we can calculate the strength of the activity at all grid points in the brain. The position corresponding to the maximum strength is assumed to be the source location.

### Source imaging via partial least squares

**Figure 1:**
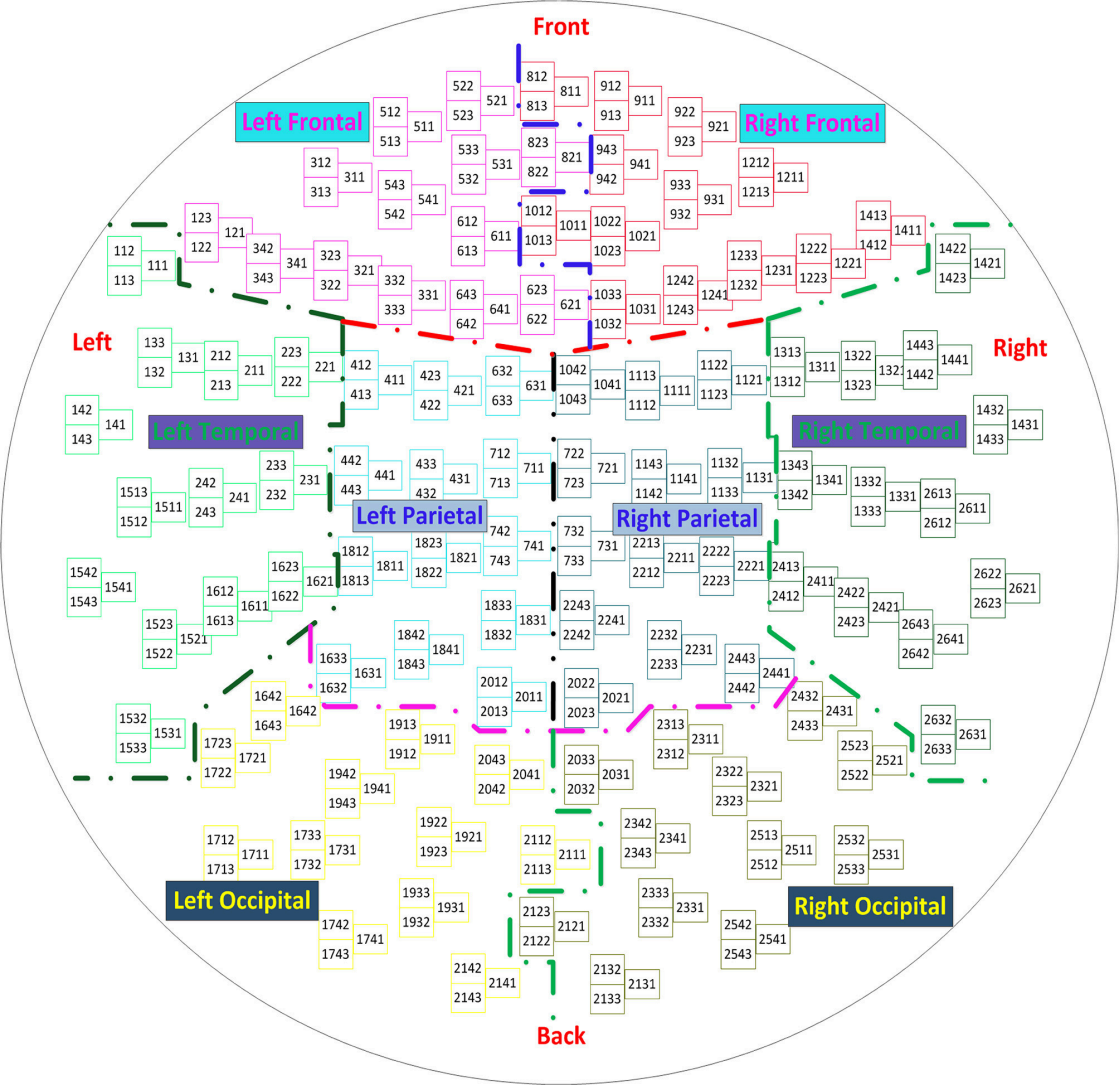
Layout of the sensor elements. The helmet-shaped sensor array is flattened into a plane, and the 306 sensor channels are divided into eight brain regions: frontal lobes (left and right), temporal lobes (left and right), parietal lobes (left and right), and occipital lobes (left and right).

The source imaging process includes four parts: head model construction, forward solution, inverse solution, and source display. The aim of this paper is to improve the localization accuracy by optimizing the inverse solution with minimum variance beamforming and PLS analysis. With the selection of the input variables ***X*** and the response variables ***Y***, the PLS analysis generates multiple variants in different application scenarios. Recent studies (Sekihara et al., 2002b; Brookes et al., 2007; Hu et al., 2017) ignore the correlations between different brain regions when reconstructing the input matrix ***X*** in the MEG source imaging. Since PLS is a supervised learning, the first step in using this method is to divide all samples into multiple classes. In the present study, each channel is considered as a sample for the input matrix ***X***. All MEG sensors are then classified to integrate the PLS method into brain source imaging. Figure 1 shows the layout of all the MEG sensors, which are divided into eight brain regions: frontal lobes (left and right), temporal lobes (left and right), parietal lobes (left and right) and occipital lobes (left and right). This classification of brain regions refers to the standard brain regions provided by Elekta Neuromag MEG. Thus, the full set of samples is divided into eight classes according to the distribution of brain regions of the sensors. The ***Y*** matrix is then generated according to these classes.

The MEG recordings were acquired inside a magnetically shielded room, with 306 channels in total, using a helmet-shaped whole-head system (VectorView, Elekta Neuromag Oy, Finland) comprising 102 locations in triplets. The system included one magnetometer and two orthogonal planar gradiometers. To compute the forward solution, we used a realistically-shaped single-shell approximation for constructing a volume conduction model based on the implementation from Nolte (2003). The anatomical MRI scans of the second patient in Section Experimental Results in Epilepsy Data were used to produce a realistic head shape in all simulated data.

Next, we consider a set of *N*-dimensional samples ***X***_*M*×*N*_, where *M* represents the total number of MEG channels (306), and *N* represents the width of the time series (600). The ***Y*** matrix represents the labels for supervised learning. The ***Y*** matrix in this study directly refers to the definition of the ***Y*** matrix in the existing PLS methods that are used for dimensionality reduction and feature extraction (Baek and Kim, 2004; Sharma and Jacobs, 2011). For the PLS analysis, according to the input matrix ***X***, we define an *M*×*C* class membership matrix ***Y*** to be

(8) $$Y=\left[\begin{array}{ccc} \begin{array}{cc} \;1_{n_{1}} & \;0_{n_{1}} \end{array} & \cdots & \;0_{n_{1}}\\ \begin{array}{cc} \;0_{n_{2}} & \;1_{n_{2}} \end{array} & \cdots & \;0_{n_{2}}\\ \begin{array}{cc} \begin{array}{c} \vdots \\ \;0_{n_{C}} \end{array} & \begin{array}{c} \vdots \\ 0_{n_{C}} \end{array} \end{array} & \begin{array}{c} \ddots \\ \cdots \end{array} & \begin{array}{c} \vdots \\ 1_{n_{C}} \end{array} \end{array}\right]$$

where *n*_*i*_ is the number of samples in the *i*-th class (i.e., the number of sensors in the *i*-th brain region), *C* is the number of classes (i.e., 8), **1**_*n*_*i*__ denotes an *n*_*i*_×1 vector of all ones, **0**_*n*_*i*__ denotes an *n*_*i*_×1 vector of all zeros, and $M=\overset{C}{\underset{i=1}{\sum }}n_{i}$. A “1” in the ***Y*** matrix means that the sensor belongs to the corresponding class, while “0” means that the sensor does not belongs to this class.

**Figure 2:**
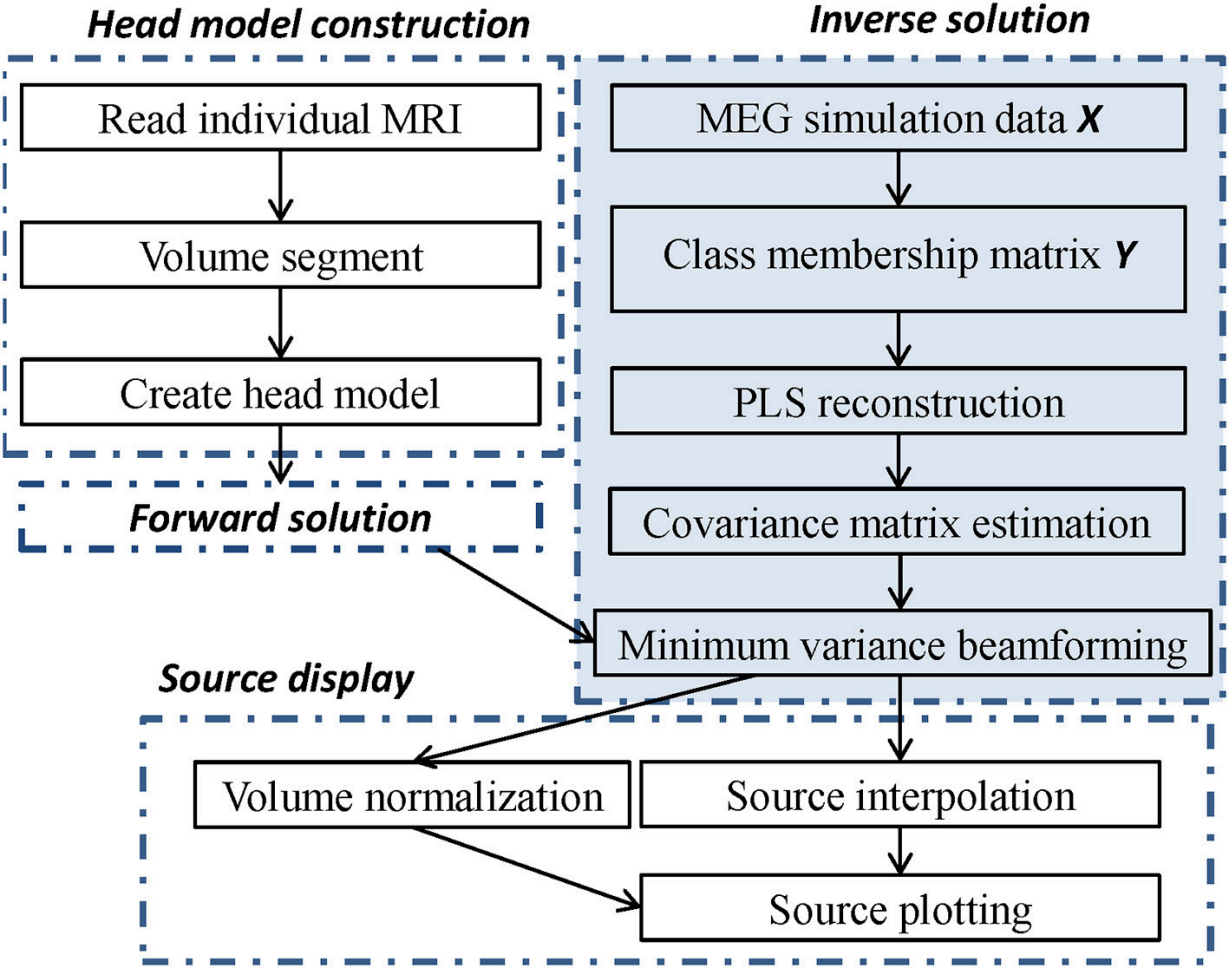
The flow chart of source imaging consists of four parts. Head model construction, forward solution, inverse solution, and source display, in which the inverse solution is modified by PLS analysis and minimum variance beamforming.

In the inverse solution, under the condition that the MEG sensor array ***X*** and the corresponding class matrix ***Y*** are known, the component matrix ***T*** and the loading matrix ***U*** are extracted using Algorithm 1, and the sensor array is reconstructed, denoted as $\hat{X}$. The reconstructed sensor array is applied to estimate the covariance matrix in formula (5), and the optimal solutions of formula (6) and (7) are obtained by optimizing the objective function (5). Using formula (7), we calculate the maximum strength and the location in the brain, which is the sought-for source. Finally, the computed source can be displayed in an individual MRI scan using an established individual head model. To clearly convey the MEG source imaging procedure, the steps are summarized in the algorithm flow chart shown in Figure 2. In the following section, we verify the feasibility of the algorithm using two different simulation sources. Three primary toolboxes, Matlab R2014a (The MathWorks Inc., Natick, MA, USA), SPM8 (Litvak et al., 2011), and FieldTrip (Oostenveld et al., 2011), are used jointly for the MEG data analysis. All source imaging algorithms are implemented based on the ft_sourceanalysis function in the FieldTrip toolkit. All parameters are optimized based on this toolkit, and the parameters of the pLCMV and mLCMV methods are the same as that of the LCMV method.

## Results

### Simulated data generation

**Figure 3:**
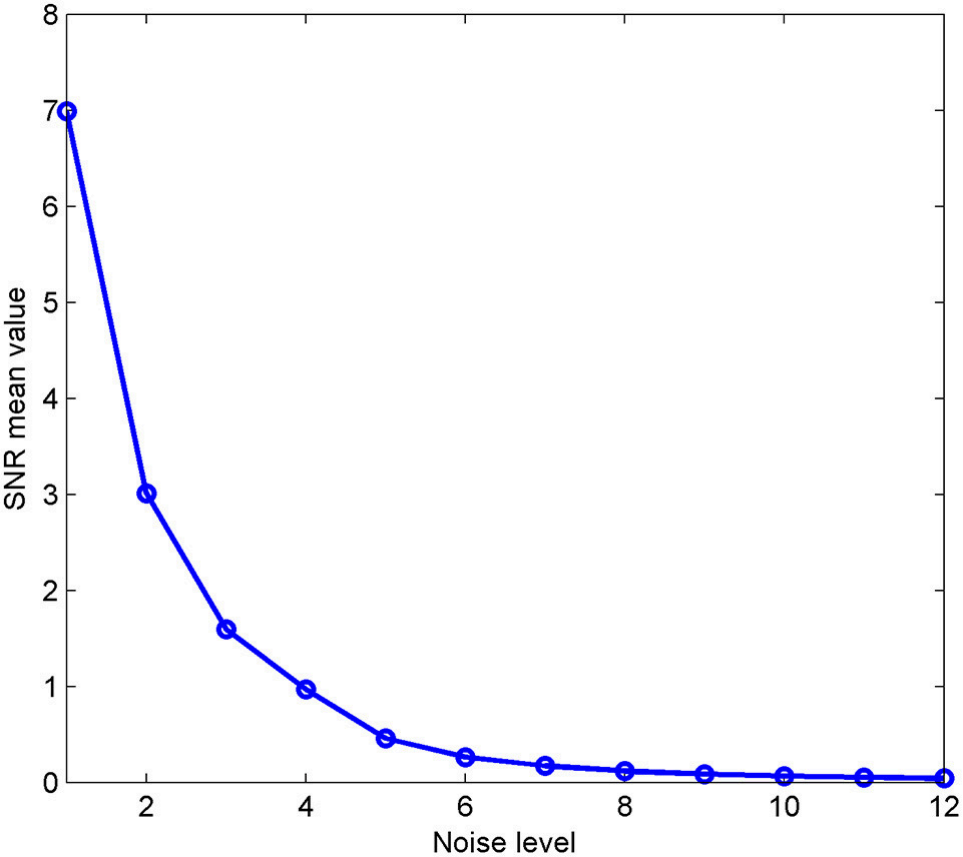
The mean signal-to-noise ratio of the simulation is given for 12 different noise levels. Fifty noise samples were generated at each level; the x-axis represents the noise level and the y-axis represents the mean value.

In view of a simulation source with explicit ground truth, we first performed experiments on simulated MEG data as follows. Source imaging is often used to find the source of event-related fields within the brain based on a task, and to locate epileptic foci. The waveforms of event-related fields and epileptiform waves are often very close to the sinc function, which is an aperiodic attenuation signal. The mathematical expression of the sinc function is described as:

(9) $$\begin{array}{r} S\;\left(t\right)\;=\;\frac{\sin\left(\pi \left(t+\tau \right)\right)}{\pi \left(t+\tau \right)\;} \end{array}$$

where τ is the translation width of the function. To show that the proposed localization algorithm can also be applied with contaminated signals, Gaussian noise was added to the time-course of the real signal. The simulated data was then generated by a sinc function plus the Gaussian noise. The noise intensity was divided into 12 levels, from weak to strong, to allow observation of the robustness of the proposed localization method. The signal-to-noise ratio (SNR) was used to quantify different noise levels, as defined in the following formula:

(10) $$SNR_{dB}=10\;\log_{10}\left(\frac{P_{A}}{P_{B}}\right)=10\;\log_{10}\left(\frac{||A|{|}_{F}^{2}}{||B|{|}_{F}^{2}}\right)$$

where *P*_***A***_ denotes the power of the synthetic sensor signals ***A***, *P*_***B***_ is the power of the background noise, and ||•||_*F*_ represents the Frobenius norm of a matrix or vector. For each level of noise, 100 Gaussian noise samples were generated randomly. The mean of these SNR values are shown in Figure 3. These SNR values decrease from a maximum of 6.990 to a minimum of 0.043 with the changes in the noise level.

In this study, the time duration of all the simulated MEG data is 600 ms, and the sampling rate is 1,000 Hz. The planar gradiometers were used to localize the sources. The source space was based on a subject's realistic head shape, which was modeled as a single shell based on a magnetic resonance imaging scan. For a realistic head model, the brain space was partitioned into a three-dimensional grid with millimeter resolution, including 3,704 points in total. Each point could be regarded as a source location. The MEG sensor arrays were implemented through the ft_dipolesimulation function in the Fieldtrip toolbox, based on the previously described synthetic signals for the location.

### Experimental results in simulated data

In this section, we first verify the source imaging algorithm through the experiments based on simulated data, generated by a sinc function plus Gaussian noise. The proposed method is compared with three well-known beamforming methods: linearly constrained minimum variance (LCMV) (Van Veen et al., 1997), dynamic imaging of coherent sources (DICS) (Groß et al., 2001), and modified LCMV with iterative matrix decomposition (mLCMV) (Hu et al., 2017). Because the new method combines PLS with beamforming, pLCMV is regarded as an acronym of the new method. Spatial accuracy is used to evaluate the results of source imaging, with the evaluation index defined as:

(11) $$\begin{array}{r} Location\;error=\sqrt{||\gamma -\hat{\gamma }|{|}_{2}} \end{array}$$

where ||•||_2_ represents the two-norm operator, γ is the spatial location of the real source, and $\hat{\gamma }$ is the spatial location of the source estimated by the localization algorithm. A smaller location error corresponds to a higher spatial accuracy.

**Figure 4:**
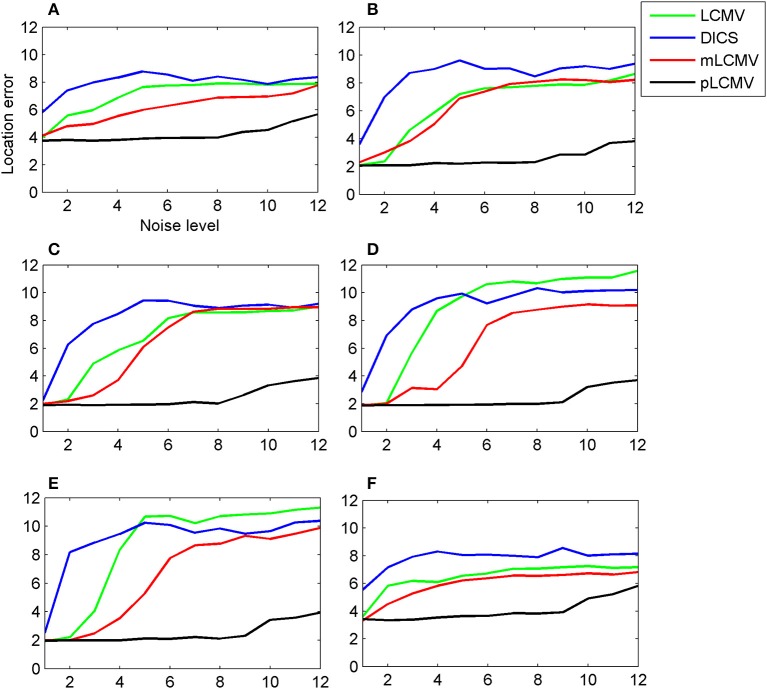
For a simulated data generated by an aperiodic signal (sinc) plus the Gaussian noise, the spatial accuracy of the proposed approach (pLCMV) was compared with that of the other three approaches (DICS, LCMV, mLCMV). The x-axis of each plot represents the noise level, and the y-axis represents the location error. Each plot shows a comparison of the localization results of the four approaches based on simulated data generated for six different locations of the brain. The six locations were the left mesial temporal lobe **(A)**, right lateral temporal lobe **(B)**, right frontal lobe **(C)**, right occipital lobe **(D)**, right parietal lobe **(E)**, and right mesial temporal lobe **(F)**. Plots **(A)** and **(F)** represent deep sources; the rest represent shallow sources.

We chose six sources to construct the simulations. The spatial locations of the six sources were represented in the Neuromag coordinate system as {(−29, 11, 38), (67, 11, 30), (59, 43, 70), (59, −53, 54), (67, −29, 86), (35, 11, 38)} mm. The six locations were in the left mesial temporal lobe, right lateral temporal lobe, right frontal lobe, right occipital lobe, right parietal lobe, and right mesial temporal lobe. The first and last were deep sources, the rest were shallow sources. Figure 4 shows that the localization results using the proposed method (pLCMV) were obviously better than those obtained from the other three methods (LCMV, DICS, and mLCMV). The spatial accuracies on the localization results using the four methods became lower as SNR value became smaller. The spatial accuracies of the three alternative methods (LCMV, mLCMV, DICS) were very similar with the increase of noise. Thus, the proposed method had the highest spatial accuracy, and that the spatial accuracy was affected little by the noise level.

**Figure 5:**
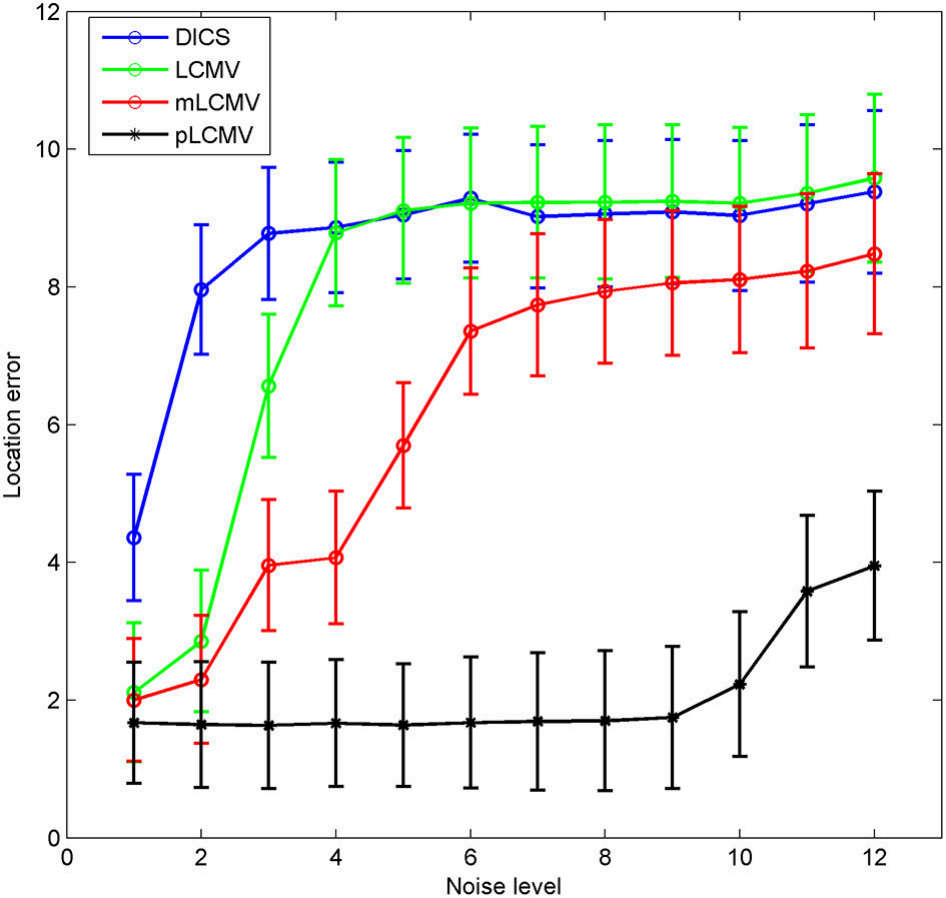
Comparison of the spatial accuracy of the four methods. The x-axis of each plot represents the noise level, and the y-axis represents the mean value ± standard deviation of the location error, based on 309 simulations. Each simulation was generated by a sinc function and Gaussian noise.

Although the **six** locations covered the major regions of the brain, these locations cannot represent all possible grid points in the brain. To obtain more representative results, 309 locations were selected from the 3,704 points of the grid, by starting from the **first** point and using a step length of 12. As in the previous set of experiments, the real signal (a sinc signal) and Gaussian noise were used to generate 309 simulations for these 309 locations. For these simulations, the location errors of the **four** localization methods (LCMV, DICS, mLCMV, pLCMV) were calculated. Figure 5 shows the mean value and standard deviation of the spatial accuracy of source localization for the 309 simulations generated using the sinc function. The localization results for the mLCMV method were better than those for DCIS and LCMV, and the difference in source localization between LCMV and DICS was not obvious. Also, the spatial accuracies on the localization results using the **four** methods became lower as SNR value became smaller, and the proposed method had the highest spatial accuracy.

### Experimental results in epilepsy data

By adding a Gaussian noise to the source signal, the experimental results show that the proposed method was effective and had the highest spatial accuracy in simulated MEG data. However, an actual MEG signal is often disturbed by noise of many complex origins, such as breathing, heart beats, eye movements, small movements of the facial muscles and so on. In the following experiments, we further verify the proposed method in a real MEG dataset with focal temporal lobe epilepsy (TLE). A total of 10 patients with medically refractory TLE were obtained retrospectively. All clinical characteristics of the patients are described in Table 1. These patients were diagnosed as focal unilateral TLE by a comprehensive preoperative assessment, including seizure history and semiology, neurological examination, 3-Tesla magnetic resonance imaging (MRI), scalp electroencephalography, invasive electroencephalography. All patients were from Xuanwu hospital in Beijing and underwent anterior temporal lobectomy including hippocampus (Schaller and Cabrilo, 2016). The results of at least **1** year follow-up indicated that these patients achieved seizure free status (Engel class IA). Written informed consent was obtained from each participant. The study was performed under a protocol approved by the medical ethics committee of Xuanwu Hospital of Capital Medical University.

**Table 1:** Clinical characteristics of the patients.

**Patient no**.	**Age (years)**	**Seizure duration (years)**	**MRI**	**Spike number**	**Preoperative assessment**	**Pathology**
1	16–20	8	HLC	45	LT	FCD
2	16–20	9	RHS	37	RT	FCD, HS
3	20–25	17	LHS	23	LT	FCD, HS
4	26–30	5	LHS	18	LT	FCD, HS
5	26–30	14	Normal	42	RT	FCD
6	20–25	14	HRH	38	RT	FCD, HS
7	30–35	11	Normal	46	RT	FCD
8	20–25	20	LHS	32	LT	FCD, HS
9	30–35	17	ARH	27	RT	FCD
10	36–40	16	Normal	25	RT	FCD

MRI indicates magnetic resonance imaging; M, male; F, female; LT, left temporal; RT, right temporal; LHS, left hippocampal sclerosis; RHS, right hippocampal sclerosis; HRH, hyper T2 in right hippocampus; HLC, hyper T2 in left temporal cortex; ARH, atrophy in right hippocampus; BHS, bilateral hippocampal sclerosis; FCD, focal cortical dysplasia; HS, hippocampal sclerosis.

**Figure 6:**
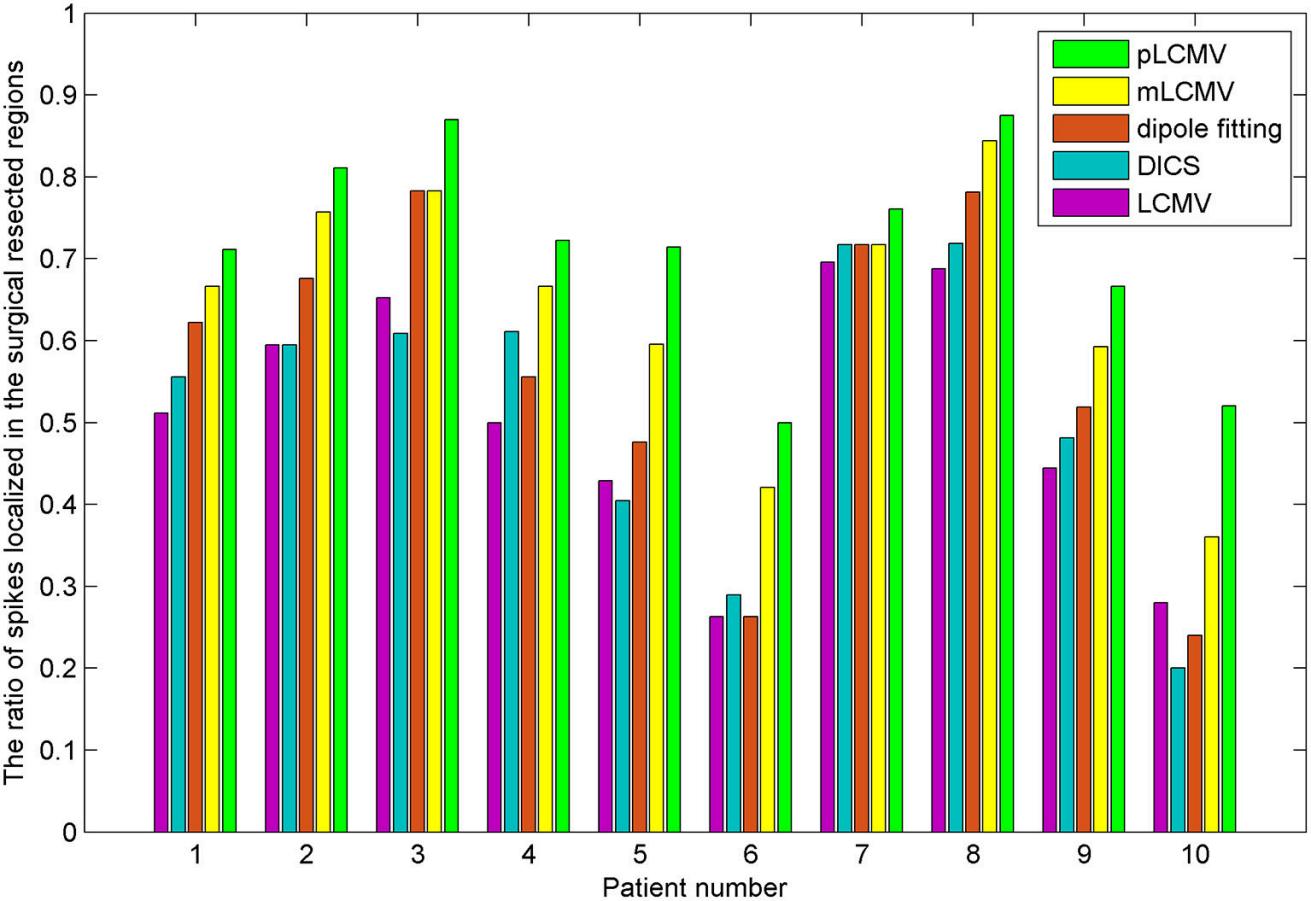
Comparison of localization results obtained for patients with TLE using the proposed method (pLCMV) to those obtained using dipole fitting, LCMV, DICS, and mLCMV methods. The x-axis represents the patient number, and the y-axes represent the ratios of the number of spikes localized in the surgical resected region to the total number of spikes.

**Figure 7:**
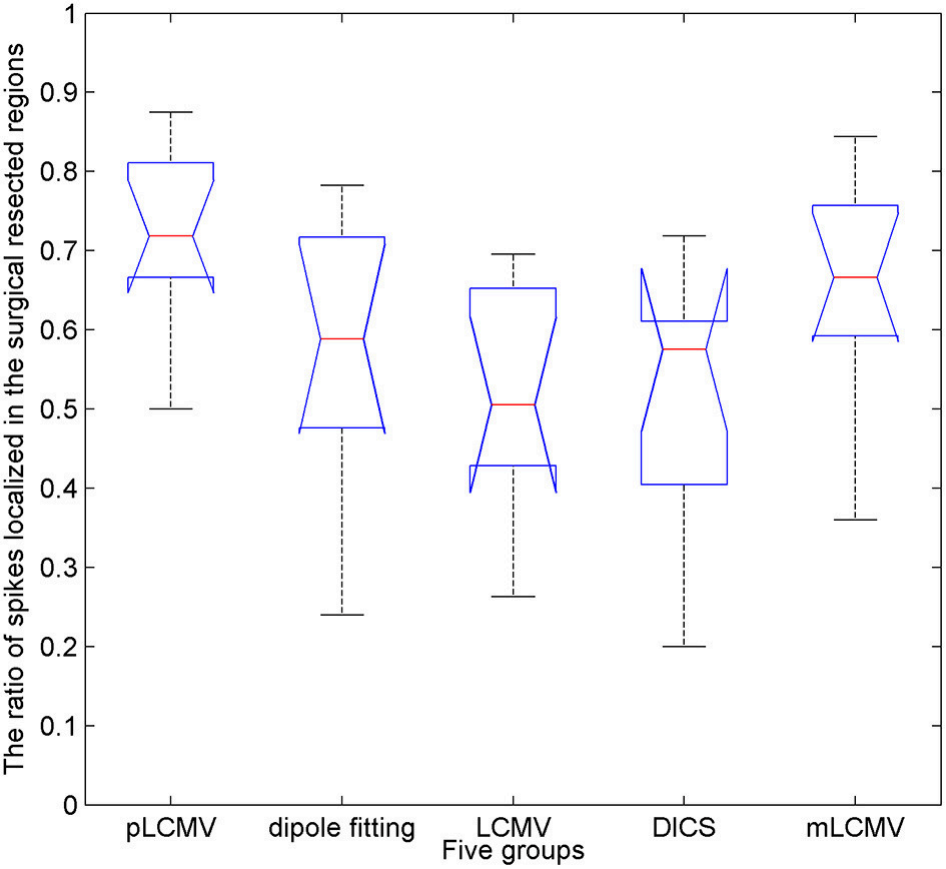
Significance test of mean difference of the five groups of localization results using analysis of variance (ANOVA). The x-axis represents the five groups, and the y-axes represent the ratios of the number of spikes localized in the surgical resected region to the total number of spikes.

The epileptic spikes were visually marked by **two** experienced clinical epileptologists in the MEG signals. The MEG data with a spike was localized using the proposed method and the dipole fitting. The dipole fitting method here is performed using the software provided by the MEG and is widely recognized in clinical epilepsy localization. **One** spike in this study yields a localization result. The localization results of all epilepsy patients were checked by the clinical epileptologists. We counted the number of those spikes in the resection region for source localization results. Figure 6 shows a comparison of localization results obtained for patients with TLE using the proposed method (pLCMV) with those obtained using the dipole fitting, LCMV, DICS, and mLCMV methods. The ratios of the number of spikes counted in the resection region to the total number of spikes in each patient are shown in Figure 6. The localization results of most spikes should appear in the surgical resection region based on preoperative assessments, pathological findings, and postoperative follow-up results. Figure 6 shows that the dipole fitting method is not always effective for finding epileptogenic zones, especially in the 6th and 10th patients. The ratios of the number of spikes localized in the surgical excised region to the total number of spikes using the proposed method were highest compared with those ratios of that using the other **four** methods: dipole fitting, LCMV, DICS, and mLCMV. The analysis of variance (ANOVA) was used to further compare the mean difference of the **five** groups of localization results. Figure 7 shows significance test of mean difference of the **five** groups using ANOVA. The localization results for the mLCMV method were better than those for dipole fitting, DCIS, and LCMV, and the difference in source localization between dipole fitting and DICS was not obvious. The localization results using the proposed method in these patients are more consistent with the clinical evaluation. The proposed method may provide a new imaging marker for localization of epileptogenic zones.

## Discussion and conclusions

MEG is a non-invasive type of preoperative examination and therefore plays an indispensable role in the localization of epileptogenic foci in epilepsy patients. Because the locations of epileptogenic zones estimated using the current localization methods are not always accurate, MEG examination results are sometimes questioned by clinical epileptologists (Englot et al., 2015). A number of source imaging algorithms for MEG recordings have been proposed and successfully applied for several purposes, such as localization of epileptic foci (Bast et al., 2004; Sutherling et al., 2008; Englot et al., 2015). In this work, PLS analysis was used in combination with minimum variance beamforming to reconstruct sensor arrays and locate sources. As is widely known, beamforming techniques play an important role in source imaging. LCMV is a typical representative of time domain beamforming, and DICS is a representative of frequency domain beamforming. These two methods have achieved effective localization results and have been successfully applied in many fields (Hoogenboom et al., 2006; Van Essen et al., 2013). The most recent literature shows that a modified LCMV (mLCMV) source imaging algorithm has been proposed, and has achieved good spatial accuracy in deep source imaging (Hu et al., 2017). Therefore, to test the effectiveness of the proposed algorithm (pLCMV), we compared it to LCMV, DICS, and mLCMV. The single-shell approximation technique was used as a forward method to construct a volume conduction model in this study. A proper head volume conductor in the forward method is helpful to improve the spatial accuracy of source imaging. Many improved forward models have been proposed for accurate source analysis and connectivity measures (Vorwerk et al., 2014; Neugebauer et al., 2017). Taking into account the cerebrospinal fluid and distinguishing between gray and white matter are effective in head volume conductor modeling. The improved forward models should be combined with the proposed method in future study, and may significantly improve the spatial accuracy of source imaging.

In conclusion, we designed a new method that combines partial least squares analysis of MEG arrays with minimum variance beamforming to localize brain activity in simulated data and epilepsy data. Compared to that obtained using DICS, LCMV, and mLCMV, the spatial accuracy obtained using the proposed method (pLCMV) was highest, and the location error fluctuated little with increases in noise. For simulations generated using a sinc function plus Gaussian noise, the proposed method had the highest spatial accuracy. The spatial accuracies on the localization results using the four methods became lower as SNR value became smaller. We further verified the proposed method in a real dataset that includes the MEG recordings of 10 patients with TLE. The localization results using the proposed method are more consistent with the clinical evaluation. The proposed method may provide a new imaging marker for localization of epileptogenic zones.

We do not hypothesize that the sources should be localized in only one brain area, because the proposed method is to search for the epileptogenic zones or the sources from the whole brain. This study focuses on the discussion of single source localization based on simulated data and real dataset. The position corresponding to the maximum strength is assumed to be the source location. If we choose the top 5 or 10% maximum strength, we may be able to solve the situation of multiple source spikes or activation. The application background of this study is to solve the problem of MEG localization of the epileptogenic zone in epilepsy surgery candidates. Usually, a large proportion of those patients (epilepsy surgery candidates) have single source (Englot et al., 2015; Nissen et al., 2016). At present, most epileptic experts believe that only one source is localized from a spike with MEG recordings (Englot et al., 2015). In addition, multiple sources can also be localized by single source localization for epileptic patients with multiple lesions. Therefore, the proposed method does not highlight the assumption of single source localization in this study. We will continue to study the multiple sources localization in future research.

### Limitations

In order to implement the PLS method, the array of sensors was divided into eight classes according to the standard brain regions provided by Elekta Neuromag MEG in this study. The ***Y*** matrix is then generated according to these classes. In fact, it is not necessary to divide all sensors into 8 classes. It is possible that 4, 12, or even 16 classes could be used to localize the sources using the PLS method. In future work, we can examine the effect of different class numbers on the spatial accuracy of source imaging. Since adjacent sensors may be divided into two different classes, this may be a challenge for PLS method to extract the components of MEG data. In future work, the proposed method should be verified in more types of epilepsy, such as frontal lobe epilepsy, insular epilepsy, and occipital lobe epilepsy. We also hope that the effective performance of the new method can be verified in more realistic scenarios, such as locating brain tumor lesions and locating functional areas.

## Author contributions

YH, YW, and JZ: designed the research; YH: wrote the manuscript; YH and JZ: designed and implemented algorithm; YH and CY: prepared figures and tables; CY and YW: collected clinical data and preliminarily analyzed the MEG data; JZ and YW: supervised study and revised the manuscript.

### Conflict of interest statement

The authors declare that the research was conducted in the absence of any commercial or financial relationships that could be construed as a potential conflict of interest.

The reviewer YZ and handling editor declared their shared affiliation at the time of the review.
